# Supplementary material for: Trends in Sales and Industry Perspectives of Package Sizes of Carbonates and Confectionery Products
Source: Foods. 2021 May 12;10(5):1071. doi: 10.3390/foods10051071 (PMC8151470; doi:10.3390/foods10051071)
Supplement: Supplementary file 1 [file foods-10-01071-s001.zip › foods-1196692-supplementary.pdf]

SUPPLEMENTARY MATERIALS

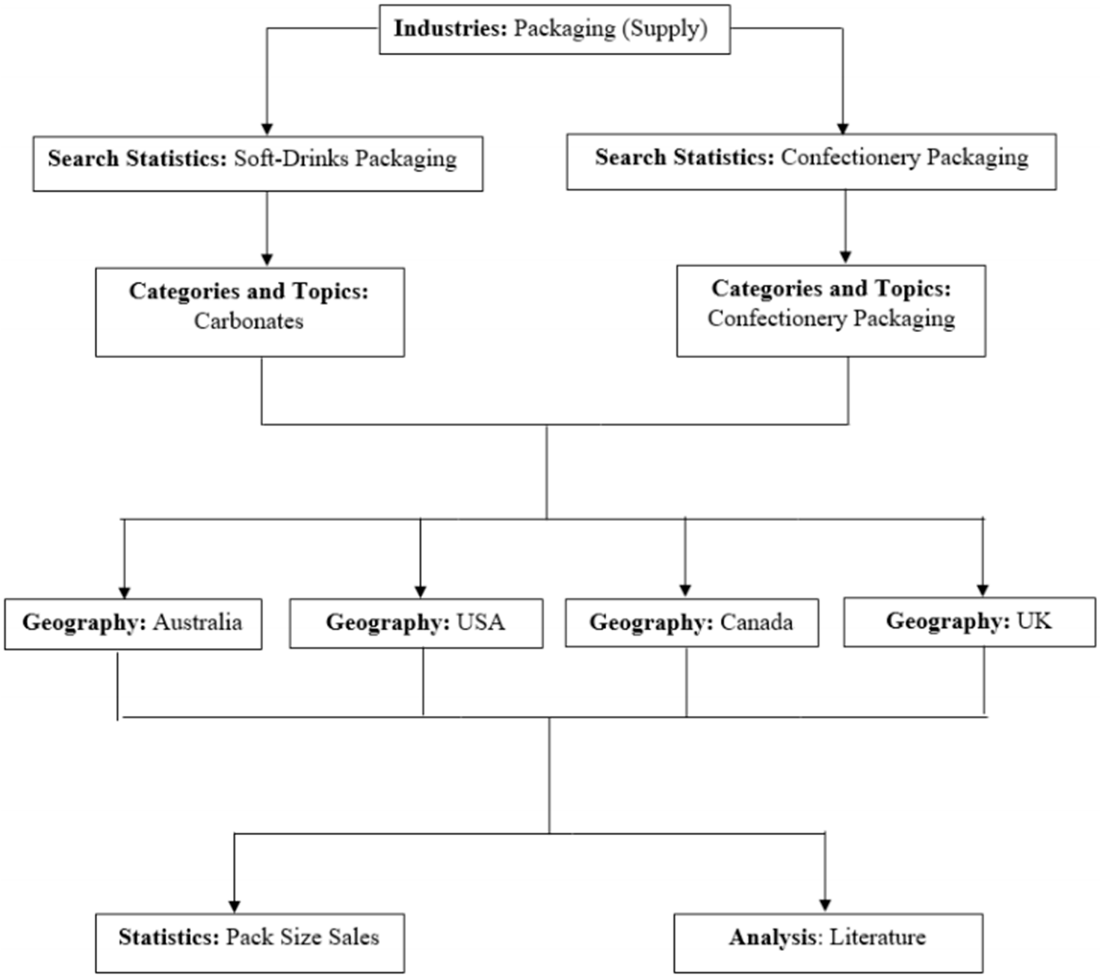

Figure S1. Search strategy and terms for sales data and qualitative data for carbonates and confectionery using the Euromonitor database[1–55]

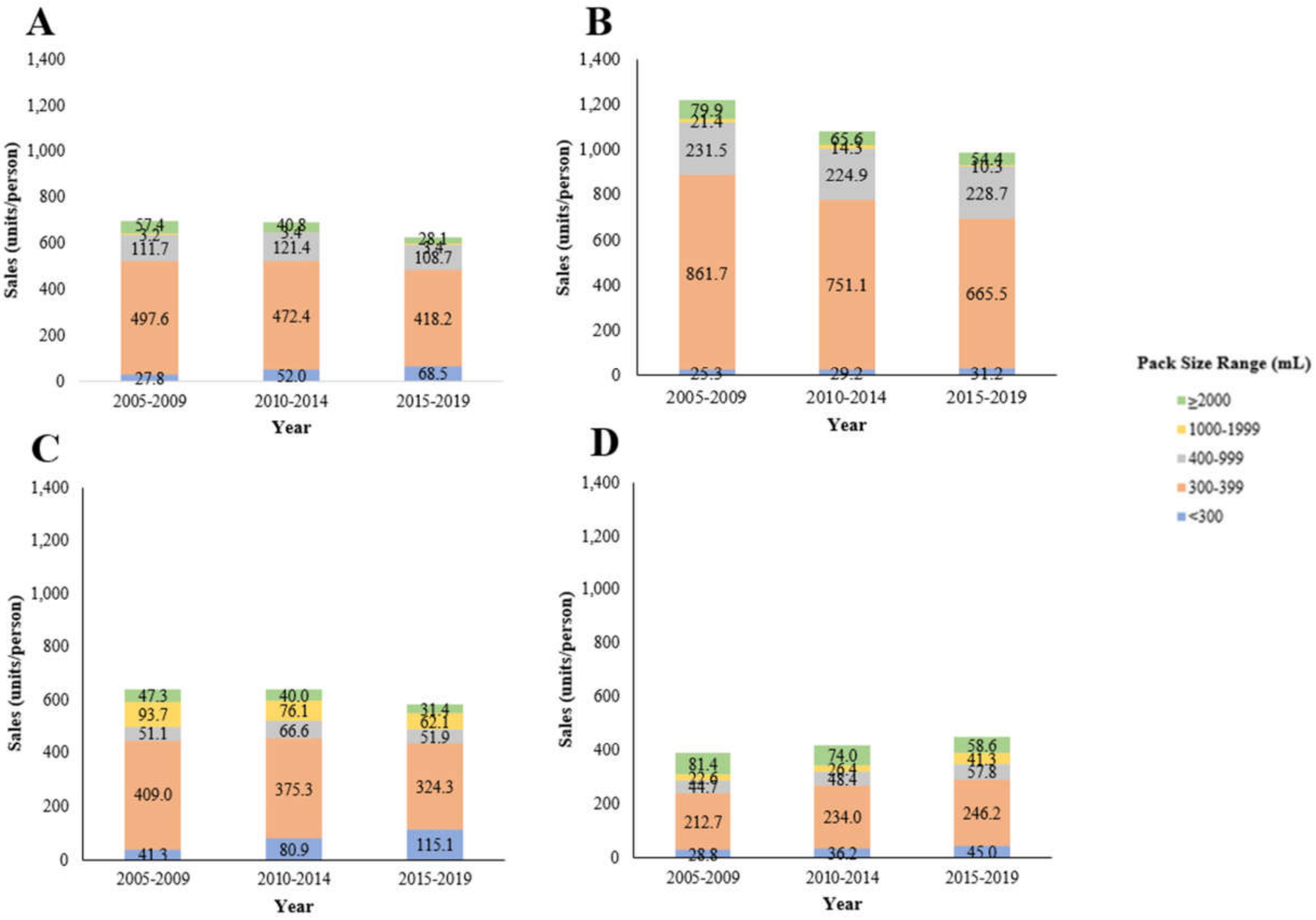

Figure S2. Retail unit volume sales per capita in carbonates over 5 years according to package size band in (A) Australia, (B) the USA, (C) Canada and (D) the UK.

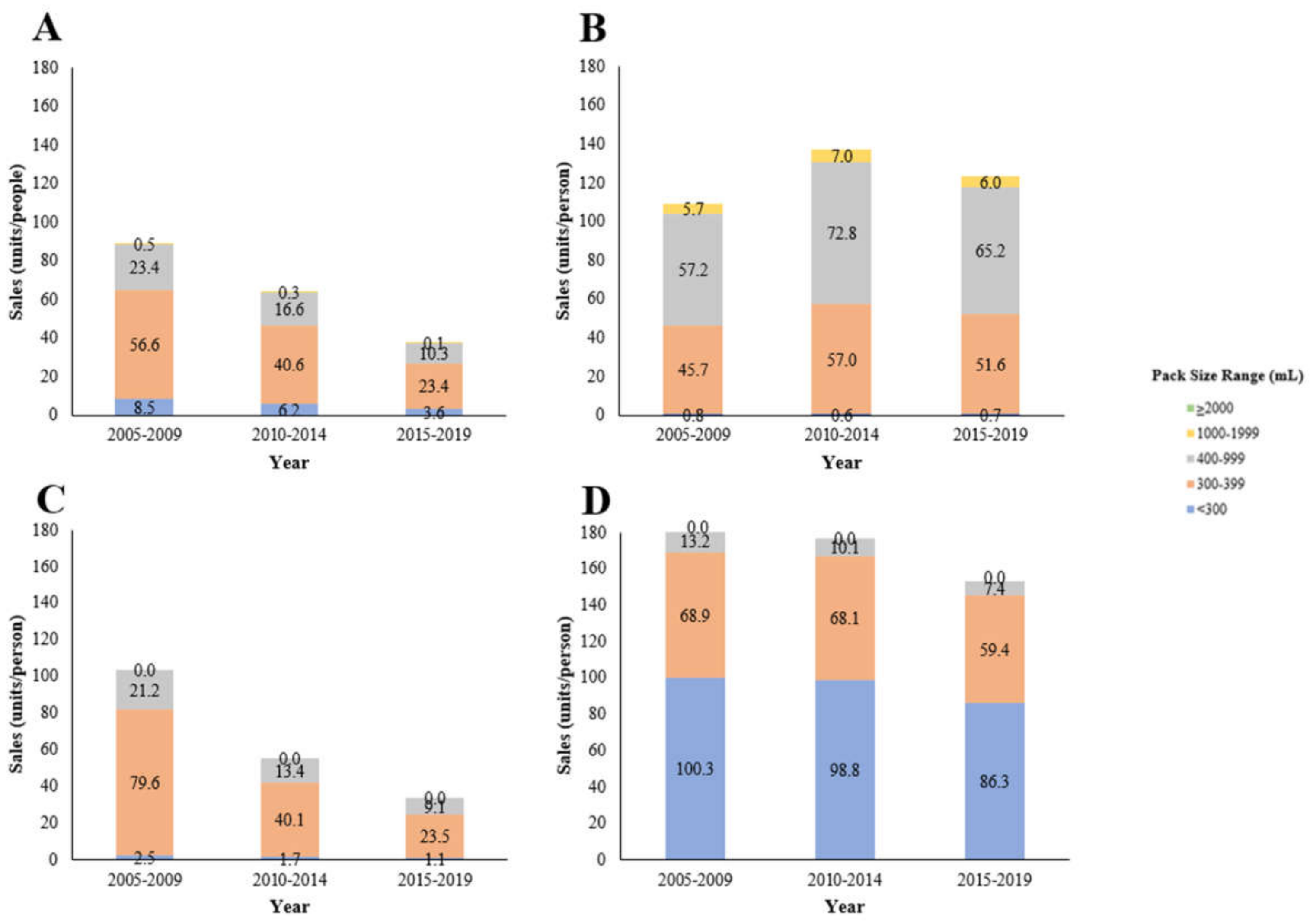

Figure S3: Foodservice unit volume sales per capita over 5 years in carbonates according to package size band in (A) Australia, (B) the USA, (C) Canada and (D) the UK.

Table S1. Search Strategy for Google Search [1]

Discretionary Group	Search Terms
**Carbonate**	(carbonates OR carbonated OR soft drinks OR soda OR Coca Cola OR Pepsico OR energy drinks) AND (pack* OR pack size OR package size OR packaged drink OR size OR larger OR smaller OR large OR mini OR small)
**Confectionery**	(confectionery OR chocolate OR Mondelez OR Cadbury OR Hershey OR Ferrero OR Nestle OR Mars OR candy OR sweets OR lollies) AND (pack* OR size OR pack size OR pack* size OR larger OR smaller OR large OR mini OR small).

[1] The discretionary topics of interest were investigated on separate searches. The search was limited to the first 100 results for screening

Table S2. Definitions for retail sales and food service sales used by Euromonitor

Sales channel	Definition
Retail/Off trade	Sales through establishments primarily engaged in the sale of goods for home use, preparation and/or consumption.<br>Includes grocery retailers, supermarkets/hypermarkets, superstores and warehouse clubs, hypermarkets, co-operatives, discounters, convenience stores, independent grocers, forecourt retailers, food/drink/tobacco specialists, booksellers and stationers, chemists and druggists, clothing/footwear/leatherwear/accessory outlets, confectionery, and news retailers (CTNs), department stores, DIY, gardening and hardware outlets, electrical, electronic and computer outlets, home furniture and furnishings outlets, jewellers, mail order, record/video games outlets, sports goods outlets, toy shops, variety stores, other food and non-food retailers.

	Includes non-store retailing such as vending, homeshopping, internet retailing and direct selling.<br>Excludes sales to/through hotels, duty-free sales, wholesale industries (including cash-and- carry) and institutional sales (sales through/to hospitals, prisons/jails, military, schools, etc, also known as contract foodservice).<br>Excludes the purchase of food and beverage products from foodservice outlets for off-premise consumption, eg impulse confectionery bought from the counters of cafés/bars.<br>Excludes the informal retail sector.
Foodservice/On-trade	Sales to – not through - foodservice establishments.<br>Includes full- service restaurants, cafés/bars, fast food outlets, 100% home delivery/takeaway, self-service cafeterias, street stalls/kiosks etc.<br>Excludes sales to/through hotels, duty-free sales and institutional sales (sales through/to hospitals, prisons/jails, military, schools, etc, also known as contract foodservice).

Table S3. Search results for industry reasons for change in package size for carbonates

Country	Year	Article Type	Title	Direction of Change	Results	Reasons for change
Australia	2009	Briefing	Soft Drinks, Weight Status and Health: A Review [55]	↑	Increased portion sizes by 2/3-fold over 50 years with disproportionate price per litre leading to consumers buying larger pack sizes.	Value for money
	2015	Webpage	Coca-Cola identifies 'portion control' among reasons for downsizing of 450ml bottle [56]	↓	Portion control achieved with 390mL bottle. Marketed as a grab-n-go size, 600mL is for extra thirst and 200/250mL is quick treat. Coca-Cola is able to deliver more options and expand SSB range.	Portion control<br>Small indulgence<br>Convenience<br>Expanding product line
				↑	Without 450mL bottle, there is no midground between 390mL and 600mL. Sales of the latter have subsequently increased.	Expanding product line
	2015	Webpage	Fizzy drinks losing their sparkle [22]	↑	Males between 14-18yo want more sugar and larger packs.	Age group
				↓	Older people want smaller drinks so replacement of 375ml can with 200 ml and smaller bottles caters to this age group. Conveniently sized packs are more popular in the market	Age group<br>Health conscious
	2016	Webpage	Coca-Cola's smaller cans may make us drink more [57]	↓	Narrative about the harms of drinking sugary drinks has intensified so Coca-Cola has opted for more profitable, smaller sized product, since packaging is such a significant contributor to price. Mini cans and bottles allow Coca-Cola to save money on aluminium and glass, most or even all of which it pockets.	Health conscious<br>expanding product line<br>Manufacturing costs
	2018	Briefing	Coca-Cola Amatil's-Select Committee into the Obesity Epidemic in Australia [58]	↓	Offer consumers a choice in sizes by offering small portion sizes to reduce sugar intake. Provide range of smaller packs in more locations for convenience. Reduced 450mL bottle to 390mL and limiting the largest single-serve size is 600mL. Increased investment in 200ml mini-can, launched new 250ml size and 390mL bottle.	Health conscious<br>Convenience<br>Expanding product line
	2018	Webpage	Big change coming to Pepsi, Coke [59]	↓	Small slimline cans for no-sugar, low-calorie Pepsi options added to range. Consumers are provided more choice and demand lower-sugar products.	Expanding product line<br>Health conscious
	2018	Briefing	Coca-Cola in Australia commits to reducing sugar by 20% by 2025 along with beverages industry [60]	↓	Smaller pack sizes to reduce sugar intake. Currently 4% energy intake in Australians is from SSBs	Health conscious
	2019			↓		Convenience

Country	Year	Article Type	Title	Direction of Change	Results	Reasons for change
		Industry Report	Global Soft Drink & Bottled Water Manufacturing [61]		Consumers turn to packaged beverages because they are portable and provide convenience for consumption away from the home. Coca-Cola introduced single-serve and multi-serve bottle and can sizes to improve sales	Expanding product line
	2020	Industry Report	Soft Drink and Pre-Packaged Food Wholesaling in Australia [62]	↓	Increasing health consciousness has limited demand for the industry's larger product segments. Less demand from café, restaurants and takeaways.	Health conscious
	2020	Country Report	Soft Drinks Packaging in Australia [63]	↓	Nexba released 450ml PET bottles in response to consumers' demand for portion control, in addition to convenience trends.	Portion control
						Convenience
USA	2005	Opinion	Packaging innovations shape soft drinks industry [64]	↓	Rising number of divorced and single consumers in the US has led to introduction of more single-serve PET bottles and multipacks on supermarket shelves as they are more convenient for individual servings. 12-ounce cans too large for one sitting, prompting Pepsi to launch 8-ounce cans.	Convenience
					Coca-Cola Company, for instance, has introduced a slim, conveniently designed 12-pack box (two cans in height and six in length) which is easy to place in the refrigerator and includes an easy-to-use snap-off top	Innovation
	2009	Opinion	Shrinking pack sizes as consumers choose quality over quantity [65]	↓	Average size of all pack types in retail carbonate drinks is shrinking due to desire for on-the-go packaging. Economic downturn has helped with producers offering smaller sizes at a lower cost. In US 20fl oz size has been replaced with 16fl oz. 1500mL size remains popular for larger family sized purchases, 300-500mL is popular single-serve choice with 813mL an average pack size.	Convenience
						Manufacturing costs
						Value for money
	2010	Opinion	Make yourself feel at home... through packaging [66]	↓	Carbonate giant Coca-Cola gives us the "fridge pack", a 12-unit enclosed carton multipack (12 oz) for fresh cans available to the entire household	Innovation
						Sharing trends
	2013	Opinion	A 12 oz Indulgence: Can Glass Counter Dwindling CSD Consumption in the US? [67]	↓	Single serve (12 oz and under) glass bottles of carbonates increased retail volumes over the last five years. May be seeking to reach a demographic of occasional consumers who enjoy carbonates in smaller, higher value quantities	Premiumisation
						Small indulgence

Country	Year	Article Type	Title	Direction of Change	Results	Reasons for change
					7.5 oz can - first introduced to the US by Coca-Cola in 2009 - was positioned as a means of portion control for health-conscious soda drinkers.	Portion control
						Health conscious
				↑	Original 6.5 oz glass bottle giving way to the 12 oz aluminium can and ubiquitous 20 oz PET plastic bottle as they provide everyday value for money.	Value for money
	2014	Opinion	Sweets & Soda: Can Carbonates Learn a Lesson from US Confectionery? [68]	↓	Coca-Cola introduced 7.5 ounce, 8-pack cans in 2011 while PepsiCo re-introduced mini cans in 2013 both in response to growing consumer propensity for portion control and reduced calories	Convenience
						Health conscious
						Portion control
					Sprite also launched an exclusive summer flavour in a 7.5 oz glass bottles to provide consumers with an occasional indulgence and convenient packaging.	Impulse buying
						Innovation
						Small indulgence
	2014	Opinion	Shrinking Packs: One Answer to Concerns over Carbonates and Expanding Waistlines [69]	↓	Concerns over sugar and calorie content have prompted shrinking of pack sizes without tinkering with product formulas; for instance Coca-cola market 7.5fl oz cans as containing 90 calories	Health conscious
	2014	Webpage	Mini Cans Bring Major Benefits to Coca-Cola Co., PepsiCo [70]	↓	20-oz -> 16-oz -> 12-oz -> more recently 8-oz. and 16-oz. Over 60% of the volume growth in brand Coca-Cola in the second quarter was driven growth in the mini can and 16-ounce immediate-consumption packages.	Innovation
						Expanding product line
	2014	Webpage	Big 3 soda makers pledge to cut drink calorie consumption by 20% by 2025 [71]	↓	Widening availability of low- and zero-calorie beverages and with drinks sold in smaller format in aim to cut beverage calories by 20% by 2025.	Health conscious
	2015	Webpage	Coca-Cola Says Its Mini Cans Are "Reinventing" The Soda Business [72]	↓	Mini can introduced as a 90calorie option and 8.5oz bottle added as an alternative to 20oz options in response to calorie concerns. It also provides moms' with a treat-size option for kids. The company is also able to charge more per litre for smaller sizes.	Health conscious
						Portion control
						Profit
	2015	Webpage	How Coke Convinced Us to Pay More … for Less Soda [73]	↓	Look of mini-cans is attractive for consumers and they are willing to may for smaller sizes.	Premiumisation
					Coca-Cola charges greater amount per litre.	Profit
					Consumers are also aware these contain less calories than the traditional size BUT seem to buy more and consume more overall.	Health conscious

Country	Year	Article Type	Title	Direction of Change	Results	Reasons for change
	2016	Webpage	Coca-Cola's clever new trick [74]	↓	In response to health concerns, manufacturers have shrunk package sizes and consequently boost profits. Consumers seem to purchase more when cans are smaller	Manufacturing costs<br>Health conscious<br>Profit
	2018	Webpage	I heard The Coca-Cola Company offers smaller package sizes. Can you tell me more? [75]	↓	Provide smaller, more convenient 7.5oz mini cans to control sugar intake and help with portion control.	Health conscious<br>Portion control<br>Convenience
	2019	Webpage	Coke shares pop ahead of one of 'most promising' new launches in decades [76]	↓	Smaller can size bringing in profits with 15% sales growth for 7.5oz cans. Offering more premium products in smaller packaging where consumers tend to consume drinks.	Profit<br>Premiumisation
Canada	2019	Webpage	Coca-Cola Canada kicks off exciting year of innovation with new mini bottle [77]	↓	Launch of Coca-Cola compact and resealable 250 mL mini bottle and 8-pack of 300ml bottles. Provides more drink options and is the first of its kind to retain carbonation in such a pack size	Expanding product line<br>Innovation
	2019	Webpage	Coca-Cola mini bottles enter Canadian market [78]	↓	Launch of Coca-Cola compact and resealable 250 mL mini bottle and 8-pack of 300ml bottles. Provides more drink options and is the first of its kind to retain carbonation in such a pack size	Expanding product line<br>Innovation
	2020	Country Report	Soft Drinks Packaging in Canada [79]	↓	Coca-Cola launches 250ml and 300ml mini PET bottles. Growing health awareness has led to downsizing of pack sizes to offer consumers greater portion control.	Portion control<br>Health conscious
UK	2011	Opinion	2010: A Turning Point for Beverage Cans in Europe [80]	↓	Smoking ban in public places has driven a decline in beverage sales through food-service outlets. On-the-go convenience with multipacks and a lower price than single unit sales.	Convenience<br>Value for money
	2013	Podcast	Beverage Cans Help Brand Owners Boost Sales Through Innovation [81]	↓	Increasing health concerns is contributing to decline in carbonated beverage sales. 250mL slim can introduced for cola carbonates which is a lower calorie alternative to standard 370mL can and presented in a slimming can type.	Health conscious<br>Innovation<br>Expanding product line
	2013	Opinion	Metal Beverage Can Proves Versatile in the Global Beverages Arena [82]	↓	Coca-Cola responds to health concerns by offering a 250ml can. The smaller, slim-line can conveys better portion control, while at the same time boosting shelf impact.	Portion control<br>Innovation
	2015	Opinion	Better than Packaging that Speaks to Consumers? Packaging that Speaks to You [83]	↓	Shrinking pack size offers portion control at a lower price point. Eases shoppers decision to treat themselves	Small indulgence<br>Small indulgence<br>Value for money
	2015	Datagraphic		↓		Value for money

Country	Year	Article Type	Title	Direction of Change	Results	Reasons for change
			Western European Beverage Packaging at a Standstill [84]		500mL PET bottle is most popular format for 2014-2019. Value for money and on-the-go suitability. Biggest volume growth for cola carbonates.	Convenience
	2018	Webpage	Good things come in small packages: The growth of smaller packaging formats [85]	↓	Various reasons for smaller pack sizes including helping consumers reduce caloric intake, in response to taxes and offering a different price point. Rise of mini-can also doubles as a mixer for alcoholic drinks. In UK, full calorie drinks are smaller to reduce sugar in response to SSB tax.	Sugar Tax<br>Value for money<br>Health conscious
	2018	Webpage	Coca-Cola to cut bottle size but increase price in face of sugar tax [86]	↓	Reduce Coke 1.75L bottle to 1.5L and increase price in the face of SSB tax	Sugar Tax<br>Profit
	2018	Webpage	The Sugar Tax [87]	↑	Increased bottle sizes of non-liable items under new tax. 1.75L bottles of Coke Zero and Diet Coke increased to 2L. Improved value for money	Value for money
				↓	Reformulation of drinks to reduce sugar content including Fanta, Sprite, Dr Pepper, Lilt and Oasis	Innovation
					Avoid changing recipe of Coca-Cola original therefore ↓ pack size from 1.75L to 1.5L	Sugar Tax
	2018	Briefing	Convenience and Impulse [88]	↓	Altered the sizes of packs to allow Coca-Cola to align price across levy liable and non-levy liable products.	Sugar Tax
	2018	Webpage	Coca-Cola to sell smaller bottles at higher prices in response to sugar tax [89]	↓	In preparation for sugar tax, Coca-Cola reduces size of bottles rather than altering recipe. 1.75L bottles will be reduced to 1.5L	Sugar Tax
	2020	Country Report	Soft Drinks Packaging in United Kingdom [90]	↓	Multipack formats are growing in favour with inflation and weak wage growth, saving money for home consumption. growth of smaller pack types across soft drinks are also for busy consumers who are health conscious, preferring to purchase 250ml and 330ml formats instead of 500ml options.	Convenience<br>Value for money<br>Health conscious
	2020	Webpage	Which beverage can sizes do Europeans prefer? [91]	↓	150ml, 200ml and 250ml slim cans are growing in importance for different kinds of drinks. These sizes appeal particularly to a younger target group as they are seen as a modern and innovative pack. On-the-go sizing for convenience as well as smaller packaging for health reasons. Various other can sizes are to be found in Europe, ranging from only 150ml up to 1 litre to suit consumer and company	Innovation<br>Age group<br>Convenience<br>Health conscious<br>Expanding product line
North America	2009	Opinion		↓		Convenience

Country	Year	Article Type	Title	Direction of Change	Results	Reasons for change
			Convenience and portability drive North American beverage packaging innovation [92]		Convenience stores in particular have been the focus of increased attention from soft drinks manufacturers. Those who make purchases via impulse channels are frequently looking for products that they can consume on the go. As a result, single-serve pack sizes continued to perform well, with 16 fl oz, 20 fl oz and 24 fl oz PET bottles, and 8.5 fl oz, 16fl oz, and 24 fl oz cans	Impulse buying
	2012	Webpage	Breaking Down the Chain:A Guide to the soft drink industry [93]	↓	Taxation of SSB's in the US has led to smaller-size packaging in response to consumers' growing concerns over what they eat and drink. smaller, portion-controlled packaging limits the serving size to 90–100 calories. Overall, more variety is given to empower consumers in making their decision on which SSB to drink depending on the occasion and lifestyle	Portion control<br>Health conscious<br>Sugar Tax
	2013	Opinion	Carbonates in the US: Is There a Threat to Packaging? [94]	↓	12 fl oz (355ml) allows a lower intake of calories and sugar while also providing a convenient recloseable solution for busy consumers.	Health conscious
				↑	the launch of different pack types and, most importantly, pack sizes will allow brand owners to reach out to a wider group of consumers and cater for various needs.	Expanding product line
	2014	Webpage	Could Smaller Servings Boost Margins For Coca-Cola and Pepsi, Amid Soda Slowdown? [95]	↓	60% market growth was attributed to sales of mini-cans for both Coca-Cola and Pepsi as consumers look for lower calorie consumption. Could also come from increasing the intake of occasional impulse buyers, who might be inclined to buy mini cans rather than the bulky multipacks. Companies target occasional customers to increase net profits due to higher unit price	Impulse buying<br>Profit<br>Health conscious
	2016	Webpage	Trends in the carbonated soft drinks market [96]	↓	↓ in household size= greater demand for smaller product packs as well as higher-quality, value-added goods. Innovation in smaller portion sizes allows consumers to enjoy the indulgence associated with CSDs but in a more controlled way. Provides on-the-go consumers an easy-to-carry option with less of a sugar hit than a larger product.	Premiumisation<br>Age group<br>Small indulgence<br>Innovation<br>Convenience<br>Health conscious
	2019	Webpage	Consumers driving shift to smaller pack sizes-Pepsico CEO [97]	↓	Smaller formats have higher per-litre prices and grow sales in value terms in the face of falling volumes	Profit
	2020	Webpage	How much sugar is in Coca-Cola? [98]	↓	Making smaller, more convenient packages to control sugar intake. Around 44% of SSBs come in convenient packages of 250 mL (8.5 oz.) or less.	Convenience<br>Health conscious

Country	Year	Article Type	Title	Direction of Change	Results	Reasons for change
Multi-national (US/Canada/UK)	2009	Opinion	Carbonates in multipacks – the battle between enclosed cartons and shrink wrap [99]	↓	Higher profit margin with multipack vs several small containers. Standard 330mL is more popular since introduction of multipacks as it provides consumer better value for money, appealing to cost-sensitive consumers and reduces purchasing occasions for convenience. Specifically, for US, Coca-Cola developed 'fridge-pack' for American refrigerators	Convenience<br>Profit<br>Value for money<br>Innovation
	2011	Opinion	Falling Carbonate Sales Stalls Beverage Can Position [100]	↓	Smaller-sized cans to aid portion control have been popular despite declining carbonate sales	Portion control
	2014	Opinion	New Global Briefing: Beverage Growth Supported by Refreshing Packaging Developments [101]	↓	Reduced can size provides affordability and lower calorie consumption. Coca-Cola introduced 250mL slim can as alternative to 330mL can.	Value for money<br>Health conscious
	2017	Podcast	How can packaging innovation support health trends in soft drinks? [102]	↓	Growing concerns over calorie and sugar intake is particularly evident in UK and US. Introduction of Dr Pepper 213mL pack type which is the right size for portion control.	Portion control<br>Health conscious
Multi-national (Global)	2009	Opinion	What's happening in soft drinks packaging [103]	↑	Growth in larger pack sizes driven by a move to economy products.	Value for money
	2015	Briefing	Where Are Our Calories Coming From? Actions Being Taken to Improve Nutrition [104]	↓	Soft drinks can be part in a balanced diet if lifestyles are healthy and active. Companies have committed to providing new variants and smaller packaging sizes	Health conscious<br>Portion control<br>Expanding product line
	2016	Opinion	Smaller is Better as Global Packaging Growth is Shaped by Variation in Pack Sizes [105]	↓	Reducing the pack size has proven valuable to enable consumers to better measure and regulate their soft drink and calorie intake. Specifically, in 2015, Coca-Cola Canada replaced 591mL → 500ml PET bottle and 355mL → 310ml slimline can. In Europe, 2L and 1.5L bottles have been reduced 1L and 1.25L to suit smaller households and regulate their consumption. Portion control and regulating sugar intake are also pertinent trends to shift consumer purchases in smaller quantities. Government initiatives to reduce sugar intake and obesity coupled with affordability further strengthens success of smaller pack size trend.	Health conscious<br>Portion control<br>Value for money
	2019	Opinion	How the Healthy Living Trend is Shaping	↓	0-300ml size range is the most dynamically performing in retail carbonate sales over 2017-2018. This is reflective of moderating intake of sugary carbonates and regarding it as an indulgence.	Small indulgence<br>Health conscious

Country	Year	Article Type	Title	Direction of Change	Results	Reasons for change
			Consumers' Packaging Choices [106]			

Table S4. Search results for industry reasons for change in package size for confectionery

Country	Year	Article Type	Title	Direction of Change	Results	Reasons for change
Australia	2015	Web Page	Shrinking pack sizes [30]	↓	Cadbury Dairy Milk reduced the size of the Freddo Frog from 15g to 12g. The family chocolate block was also reduced in size from 250g to 200g in 2009 and then increased again to 220g in 2013. Companies needed to shrink the pack size to make up for higher manufacturing costs	Manufacturing costs
	2018	Web Page	Frequently Asked Questions: Allen's Lollies [107]	↓	Killer Python sizes were decreased in 2014.	Portion control
	2019	Web Page	Cadbury to reduce the size of its chocolate blocks [26]	↓	In 2015 the standard Cadbury 220g block was reduced to 200g. Cadbury decided to reduce the block further to 180g. Cadbury Dairy Milk Picnic will also go from 180g to 170g and Old Gold will go from 200g to 180g. Mondelez states increased input costs were behind the reduction in size.	Manufacturing costs
				↑	Cadbury bubbly blocks will increase from 155g to 160g.	Value for money
	2019	Industry Report	C1182: Chocolate and Confectionery Manufacturing in Australia [108]	↓	Mondelez has decided to reduce the size of several of its chocolate bars.	Profit
						Manufacturing costs
	2019	Country Report	Chocolate Confectionery in Australia [109]	↑	Larger pack sizes are being released as share packs for sharing occasions. For example, Robern Menz reintroduced a bag format of Violet Crumble in 2019 which was previously stopped in 2010. This was reintroduced in the hopes of sharing nature of the treats leading to greater portion control.	Small indulgence
						Portion control
						Sharing trends
	2020	Country Report	Confectionery Packaging in Australia [110]	↓	Manufacturers are producing more resealable share packs for chocolate and sugar confectionery. Particularly, in chocolate confectionery, share packs are increasing for chocolate bags, pouches and tablets.	Portion control
						Sharing trends
						Small indulgence
						Health conscious

Country	Year	Article Type	Title	Direction of Change	Results	Reasons for change
					Increased purchases of smaller, single-serve portions rather than bigger portions or multi-packs due to changing consumer concerns regarding health.	Convenience
US	2014	Opinion	Guilt-Free Snacking? How Share Bags are Contributing to Confectionery Growth [49]	↑	Miniaturisation of countlines into larger sized share packs is expected to increase value sales.	Value for money<br>Sharing trends
US	2014	Opinion	Sweets & Soda: Can Carbonates Learn a Lesson from US Confectionery? [68]	↓	Hershey Co and Mars have introduced bite-sized and miniature versions. Hershey introduced bite sized versions of its Jolly Rancher and Twizzlers brands. Mars introduced its miniaturised Starburst brand as Starburst Minis in 2013. The servings are smaller than traditional Starbursts.	Sharing trends<br>Innovation<br>Portion control
US	2014	Video	Five Trends in Snacks from the Sweets and Snacks Expo 2014 [48]	↑	Mini bite-sized products are being introduced such as York and Twix Mini's in larger share bags.	Convenience<br>Innovation<br>Sharing trends
US	2020	Country Report	Confectionery Packaging in the US [51]	↓	Mars Chocolate, Wrigley, Lindt, Ghirardelli and Ferrara Candy Company will be introducing smaller pack sizes for their products. Whilst some consumers are happy to eat a king size candy bar in one sitting, others might be reluctant to purchase such a big size due to the temptation of eating the whole thing. Mondelez announced that it aims to put 20% of its products in portion-controlled pack sizes of 200 calories or less by 2025.	Small indulgence<br>Health conscious<br>Portion control<br>Expanding product line
Canada	2020	Country Report	Confectionery Packaging in Canada [111]	↓	Stand-up pouch formats generally introduced , such as 'minis', 'bites', 'thins' and 'miniatures' with such products gain in popularity due to the relative ease of portion control and sharing e.g. Reese Miniatures Stuffed with Pieces was launched as stand-up pouches in 2018 to cater for sharing occasions. Ferrero Rocher launched a three-unit 68g pack. Hummingbird developed a smaller pack size of 28g for Ontario retailer Farm Boy to encourage impulse purchases.	Portion control<br>Innovation<br>Sharing trends<br>Encourage impulse purchase
UK	2005	Opinion		↓		Portion control

Country	Year	Article Type	Title	Direction of Change	Results	Reasons for change
			Cadbury fights obesity with portion control [112]		Cadbury introduced a 99-calorie chocolate tablet. To not compromise taste, the 99-calorie range is simply a thinner version of its regular Dairy Milk product line. This will simply allow health conscious consumers to cut calories by eating smaller portions.	Health conscious
	2006	Opinion	Minisize me: Portion control as the new path to healthy eating [33]	↓	Confectionery firms agreed to stop selling some of their king-size chocolate bars. Tempered indulgence is in response to sales declines. Hershey's released a 60-calorie Hershey's Sticks.	Portion control<br>Health conscious<br>Small indulgence
	2006	Web Page	Mars admits shrinking Maltesers and Galaxy Counter Packs was 'difficult decision' [29]	↓	The malteser brand was shrunk from 121g to 103g per bag without changing the price of the product. Toblerone was also reduced from 400g bars to 360g and 170g bars to 150g by brand Mondelez.	Manufacturing costs
	2013	Opinion	Manufacturers Look to Packaging to Up Impulse Purchase of Miniaturised Foods [37]	↓	Line and brand extensions and different pack sizes are being widely used to achieve this via the launch of miniature product versions with Dairy Milk Bubbly mini tablets made available in a 90g flexible plastic.	Small indulgence<br>Portion control<br>Expanding product line
				↑	M&M's are being offered in larger share pack pouches, 315g.	Sharing trends
	2013	Podcast	Sizing as a way to sustain pack growth in snacking products [38]	↑	Share packs are increasingly common. For example, 150g-250g large plastic pouches performed very well in 2012 compared to the standard pack size of 35g-45g for Maltesers and M&Ms. In 2013, M&Ms also released a larger pouch version of the snack, being 315g.	Value for money<br>Sharing trends
				↓	Bite size packaging also on trend- smaller package sizes of pastilles and jellies were seen such as Jelly Belly's.	Portion control
	2014	Briefing	Global Food Packaging: Nurturing Future Sales Growth in Foods Through Packaging Innovation [35]	↓	Portion sizing often results in higher profit margins for brand owners, while the strategy also responds to consumers' desire to continue to treat themselves while better controlling calorie intake e.g. Dairy Milk Bubbly mini tablets were made available in 90g flexible plastic.	Small indulgence<br>Profit<br>Portion control
				↑	M&Ms relaunched in a 315g plastic sharing pack pouch.	Sharing trends
	2014	Opinion	How flexible packaging enables product	↑	Bagged softlines brand M&M's launched a "More to Share" 315g share pack plastic pouch in 2013	Value for money<br>Sharing trends

Country	Year	Article Type	Title	Direction of Change	Results	Reasons for change
			affordability in confectionery [36]			
	2015	Opinion	Better than Packaging that Speaks to Consumers? Packaging that Speaks to You [83]	↓	Major food and drink brand owners are shrinking pack sizes to offer greater portion control and a lower price point.	Portion control
						Value for money
					In 2015, countline manufacturer Nestlé SA chose to give British consumers various reasons or excuses to have a KitKat bar, such as "rainy day", "me time", or "YouTube Break" easing their decision to treat themselves to their product against the backdrop of a health and wellness trend.	Health conscious
						Small indulgence
	2015	Opinion	Pack Downsizing strategy to meet snacking demand- flexibles show the way [52]	↓	Mondelez International Inc introduced Cadbury Dairy Milk Lu and Ritz countlines range in 35g flexible plastic packs in 2014 as a way to innovate while keeping costs under control and in line with consumers who increasingly watch their spending and prefer to treat themselves to a smaller quantity	Expanding product line
						Innovation
						Value for money
						Small indulgence
	2015	Opinion	Window of opportunity for confectionery and bakery packaging in Europe [113]	↓	Ferrero produced its countline brand Kinder Bueno as a miniature chocolate as calorie intake being of increasing concern in Europe	Health conscious
					Lindt also produced a Lindor range packaged in 200g folding carton. It is popular in the UK as a product suggesting a personal treat or to be shared any time	Small indulgence
						Sharing trends
	2016	Briefing	Sizing Strategies in Global Food Packaging: Smaller Packs for Higher Value [31]	↓	Snacking goes hand in hand with consumers feeling more and more time deprived and eating on the go. This is related to rising health awareness among consumers in mature economies and brand owners responding with pack formats that suggest greater portion control over calorie intake.	Portion control
						Convenience
						Health conscious
					Brand extension and smaller pack size is adopted as a way to innovate while keeping costs. In 2015, Mondelez ceased producing Cadbury Car and a Half in a 75g flexible pack, which contained 400 calories. Instead, they only offered its 45g standard	Innovation
						Health conscious

Country	Year	Article Type	Title	Direction of Change	Results	Reasons for change
					version which contained 240 calories. Further pack sizes such as 35g, 40g and 45g were also expected to take share from the standard 50g format.	
	2017	Briefing	Confectionery in Western Europe [47]	↑	Kinder launched Kinder miniature version sharing bags in the UK due to sharing and on-the-go trends.	Sharing trends<br>Convenience
	2017	Web Page	Jaffa Cakes packet size reduced in latest 'shrinkflation' move [28]	↓	Double Deckers and Picnic bars sold in multipacks are now 14% and 21.5% smaller respectively than those sold individually, while the overall pack price is unchanged. Toblerone has also spaced out its signature triangles and Mars shrank its sharing bag of Maltesers by 15%. Cadbury said rising ingredient costs and the devaluation of sterling since Brexit were making food products more expensive to make. These are the latest examples of shrinkflation- where product size reduces with consumers paying the same price.	Manufacturing costs<br>Profit
	2018	Web Page	Biscuits and chocolates take the 'shrinkflation' test [24]	↓	Compared to 2014, Snickers (4 pack) have reduced from 232g to 167g. Toblerone Milk Chocolate 200g to 150g, Twix twin bars (4 pack) 200g to 160g, Jaffa Cakes 150g to 122g, Hobnobs Milk chocolate tube 250g to 205g, Yorkie raisin and biscuit (3 pack) 160g to 132g, Kit Kat Chunky 48g to 40g, Yorkie milk chocolate 55g to 46g, Twix Funsize (bag) 320g to 275g, Twix Twin Bar 58g to 50g, Yorkie Raisin & Biscuits 53g to 46g, Mars Funsize (bag) 288g to 250g, Snickers (7 Pack) 336g to 292g, Kit Kat Chunky peanut butter 48g to 42g, Snickers Bites (bag) 136g to 119g, Toblerone Fruit & Nut 400g to 360g, Toblerone milk chocolate 400g to 360g and Kit Kat Finger Bar 45g to 42g. Manufacturers blame increased ingredient costs although also stating less calories as the reason for change.	Manufacturing costs<br>Health conscious
	2018	Web Page	Cadbury extends Darkmilk range with new pack size and flavour [32]	↓	Cadbury will introduce a 35g bar. The business will also add a salted caramel flavour Darkmilk variant, as an 85g tablet. It is believed this will help independent retailers drive impulse sales as consumers perceive better value, convenience and ease of purchase of these packs.	Innovation<br>Expanding product line<br>Encourage impulse purchase<br>Convenience

Country	Year	Article Type	Title	Direction of Change	Results	Reasons for change
						Value for money
	2019	Country Report	Sugar confectionery in the United Kingdom	↓	Strock UK launched Werther's Originals Soft Caramels in 110g and 125g sharing bags. Manufacturers are also focusing on getting a variety of pack size options, product variety and price.	Expanding product line<br>Value for money<br>Sharing trends
	2019	Country Report	Chocolate confectionery in the United Kingdom [39]	↓	Consumers are turning to high-end chocolate as they adopt a 'better but less' approach.	Small indulgence<br>Premiumisation
				↑	Mondelez introduced new sharing bags in 110g and 120g formats, with miniature version of its Cadbury's Dairy Milk Fudge, Curly Wurly and Picnic Ranges. A 440g sharing format of the Reese's peanut butter cup and Reese's Peanut Butter Cups Stuffed with Reese's Pieces.	Sharing trends<br>Expanding product line
	2019	Briefing	Chocolate confectionery in Western Europe [114]	↓	Manufacturers are overcoming a slow growth in confectionery by creating smaller and personalised pack sizes.	Health conscious<br>Small indulgence
	2020	Country Report	Confectionery Packaging in the United Kingdom [115]	↓	Smaller on-the-go packet types and sharing bags are a trend. Mondelez launched Cadbury dairy milk mini bars in a pouch, with each individual wrapped portion containing less than 100 calories.	Health conscious<br>Portion control<br>Convenience
					Wheyhey Chocolate Crispy Clusters chocolate pouches and bags (2019) come in a smaller pack with '90 calories' and 'high protein' front-of-pack claims. McVitie's Jaffa Cake Nibbles are available in a 100g sharing bag and a 39g on the go pack.	Small indulgence<br>Sharing trends
North America	2015	Opinion	"Share packs" give value for money its true meaning in snacking products [116]	↓	Twix Bites was launched in a 200g plastic pouch with a zip in the US whilst Kit Kat has appeared in a 210 g format in Canada likely in response to health and wellness trends by offering portion control.	Health conscious<br>Portion control
				↑	Making bigger pack formats available as an alternative to more standard sizes invites the shopper to use the pack for shared consumption and provides on-the go convenience	Sharing trends<br>Convenience

Country	Year	Article Type	Title	Direction of Change	Results	Reasons for change
Multi-national (Canada/US/UK)	2014	Briefing	Global Confectionery Overview: Key Categories, Countries and Trends to 2019 [117]	↑	Resealable sharing bags were launched by many brands such as Kit Kats, Reese's and Cadbury Bitsa Wispa. These command a higher price due to their larger size	Innovation
						Sharing trends
	2014	Datagraphic	Extensive Sizing Options Ensure Flexibles Succeed in Confectionery [118]	↓	Purchases of smaller flexible plastic packs have increased between 2008-2018 as it addresses search for value for money and offers small indulgence	Value for money
						Small indulgence
				↑	US has the highest absolute growth globally for larger share bags between 2013-2018 with Europe also showing increased growth.	Sharing trends
						Value for money
	2015	Video	Affordability is Key in Global Food Packaging [23]	↓	Cadbury Dairy Milk LU and Ritz Countlines were launched in a 35g flexible plastic pack in the UK.	Value for money
				↑	Larger share pack sizes are being released in NA.	Value for money
						Sharing trends
	2015	Opinion	The Good, the Bad, and the Ugly: Assessing Sugar Confectionery [119]	↑	Introduction of sharing bags to justify higher unit prices.	Profit
	2016	Video	2015 a Solid Year for Packaging, Retail Volumes Reach 3.35 Trillion Units [120]	↓	Decrease in packaging size due to snacking trend- war against sugar and increasing health consciousness. Packaging is more portable, smaller in size and offers more portion control. Small treats to be shared.	Portion control
						Sharing trends
						Small indulgence
						Health conscious
						Convenience
	2018	Briefing	Global Snacks Packaging: Staying Relevant Through Pack Sizing Strategies [27]	↓	Consumers, mainly millennials, are choosing quality over quantity in Europe and North America as they are increasingly aware of the direct correlation between lifestyle and health, and are favouring quality over quantity, 101-300g pack size is more prevalent than at a global level, but the smallest formats have the widest appeal. Confectionery leaders such as Nestle and Mars, will relaunch and repackage some of their flagship brands in mini versions.	Health conscious
						Premiumisation
				↑	Brand owners increasingly offer new pack formats between 101-300g as it suggests greater control over calorie intake. It also	Portion control
						Convenience
						Sharing trends

Country	Year	Article Type	Title	Direction of Change	Results	Reasons for change
					offers more convenient packaging, value for money and promotes shared consumption.	Value for money
				↓	Shrinkflation: product's quantity is reduced while retail unit price is constant. In 2016, Mars Inc' Maltesers plastic pouches shrank from 121g to 103g (15% reduction) in the UK. In 2017, M&M's and Minstrels decreased their pack sizes also. M&M's replaced its 141g size to 121g and 184g format to 166g. Minstrel's pouches went from 232g to 210g.	Profit<br>Manufacturing costs
Multi-national (Australia/UK)	2008	Web Page	Mars (Chocolate Bar) [121]	↓	In the second half of 2008, Mars UK reduced the weight of the regular bars from 62.5g to 58g. Sizes available of Mars bars included: miniature "Fun Size" 19.7g, "Snack Time" 36.5g, a multi-pack size of 54g, a regular sized single 58g bar and a "King-size" 84g bar. The King bar has sinced been replaced by "Mars Duo" 85g. The duo pack contains 2 smaller 42.5g bars instead of 1 large one.	Portion control
	2009				The Mars Bar size reduced from 60g to 53g in Australia and to 51g in the UK	Manufacturing costs
	2013				The 'standard' Mars bar was further reduced to 51g in the UK.	Manufacturing costs
Global	2009	Briefing	Global Packaged Food: Driving Confectionery Retail Values in an Uncertain Economic Climate [122]	↓	In the US, Hershey Bliss was launched in 2008 as a smaller size, portion-controlled luxury chocolate line. Globally Mars announced that they will shrink the size of its Galaxy chocolate bars by 17%, 150g to 125g. The reduced size of chocolate confectionery provides value for money but also for weight-conscious consumers.	Portion control<br>Value for money<br>Health conscious<br>Small indulgence
	2015	Opinion		↓		Manufacturing costs

Country	Year	Article Type	Title	Direction of Change	Results	Reasons for change
			A Bittersweet Future for Sugar Consumption in Packaged Food [25]		Mars, Mondelez Hershey and Nestle have all reduced the size of their countlines by between 10-20% usually without reducing the unit price of the products. This was also applied to Mondelez's Cadbury products. For the manufacturer, this is in response to increased cost of cocoa and for the consumer, it fits in with less calorie consumption.	Health conscious
	2019	Briefing	Global chocolate confectionery: Permitting indulgence again [123]	↓	In 2017 Mars extended the M&M’s product portfolio with Snack Mix in pocket-sized flexible plastic. Fast rising appeal of chocolate pouches and bags which typically consists of bite-sized chocolate treats as it answers demand for greater portion control when it comes to calorie and sugar intake. Suggesting shared consumption is another driver complementing this rationale, as consumers attach increasing importance to quality social time. It also provides an affordable option when purchasing discretionary choices	Portion control<br>Health conscious<br>Value for money<br>Sharing trends<br>Expanding product line
